# Impact of Host Age and Parity on Susceptibility to Severe Urinary Tract Infection in a Murine Model

**DOI:** 10.1371/journal.pone.0097798

**Published:** 2014-05-16

**Authors:** Kimberly A. Kline, Drew J. Schwartz, Nicole M. Gilbert, Amanda L. Lewis

**Affiliations:** 1 Singapore Centre on Environmental Life Sciences Engineering, School of Biological Sciences, Nanyang Technological University, Singapore, Singapore; 2 Department of Molecular Microbiology, Center for Women’s Infectious Disease Research, Washington University School of Medicine, St. Louis, Missouri, United States of America; University of Kansas, United States of America

## Abstract

The epidemiology and bacteriology of urinary tract infection (UTI) varies across the human lifespan, but the reasons for these differences are poorly understood. Using established monomicrobial and polymicrobial murine UTI models caused by uropathogenic *Escherichia coli* (UPEC) and/or Group B *Streptococcus* (GBS), we demonstrate age and parity as inter-related factors contributing to UTI susceptibility. Young nulliparous animals exhibited 10–100-fold higher bacterial titers compared to older animals. In contrast, multiparity was associated with more severe *acute* cystitis in older animals compared to age-matched nulliparous controls, particularly in the context of polymicrobial infection where UPEC titers were ∼1000-fold higher in the multiparous compared to the nulliparous host. Multiparity was also associated with significantly increased risk of *chronic* high titer UPEC cystitis and ascending pyelonephritis. Further evidence is provided that the increased UPEC load in multiparous animals required TLR4-signaling. Together, these data strongly suggest that the experience of childbearing fundamentally and permanently changes the urinary tract and its response to pathogens in a manner that increases susceptibility to severe UTI. Moreover, this murine model provides a system for dissecting these and other lifespan-associated risk factors contributing to severe UTI in at-risk groups.

## Introduction

Urinary tract infection (UTI) is one of the most common bacterial infections in humans, with an estimated annual incidence rate of nearly 13% in women [1]. Approximately 8 million outpatient visits occur each year due to UTI, costing an estimated $2 billion in annual health care costs in the United States [2]. Uncomplicated UTI are most often caused by uropathogenic *Escherichia coli* (UPEC). Complicated UTI is associated with functional or structural urinary tract abnormalities, pregnancy, or urinary catheterization. These host factors are associated with increased rates of infection with a more diverse array of organisms, including *Streptococcus agalactiae* (Group B *Streptococcus*, GBS). GBS commonly colonizes the same anatomical niches (urogenital tract, gut) as UPEC, is adept at causing infections in a variety of human tissues including the urinary tract, and has immune modulatory capabilities [3],[4],[5].

Physical, hormonal, and immunological changes that occur throughout the female lifespan are thought to put women at additional risk of bladder infection (cystitis), as well as ascending kidney (pyelonephritis) and disseminated (bloodstream) infections [6], [7], [8], [9]. For example, a number of studies show that asymptomatic bacteriuria (ASB) is common in pregnancy and is associated with higher risks of symptomatic cystitis, ascending pyelonephritis, and preterm delivery [10], [11]. In contrast, associations between ASB and UTI in non-pregnant individuals appear to be more complex, in some cases even showing a protective effect of ASB [12], [13], [14], [15]. Other studies have correlated increased parity (number of gestations) and advanced age with a greater risk of UTI [1], [7]. Indeed, pregnant women and the elderly suffer a greater risk of complicated UTI and other adverse outcomes, such as pyelonephritis, bacteremia, and urosepsis [16], [17], [18]. In addition to the influence of parity, UTI rates and bacterial etiologies differ between younger and older populations [1], [18]. Despite the unique risks of UTI to women at particular life stages, *in vivo* models of UTI almost exclusively study this infection in young nulliparous animals.

Here we examine the impact of age and parity on host susceptibility to acute and chronic UTI across a range of ages and prior reproductive experiences. We use a well-characterized murine model of UTI in female C3H/HeN mice, which have been extensively used for studies of acute and chronic UTI [4], [19], [20], [21], [22], [23], [24], [25]. UPEC colonization in this model follows a reproducible cascade of intracellular and extracellular bladder colonization events during the first 24 hours of acute infection [25], [26]. Thereafter, a subset of UPEC-infected animals experience persistent bacteriuria throughout their lifetime, displaying >10^4^ CFU bacteria in the bladder and per milliliter of urine [19]. We characterized infection dynamics for UPEC and GBS, and examined whether GBS modifies susceptibility to UPEC infection, as we have recently reported in young nulliparous mice [3]. In that study, we examined the consequence of multi-species inoculation into the urinary tract, because UTI in humans often proceeds from exposure to inherently polymicrobial bacterial community derived from the gut and/or periurethral area. We showed that GBS presence along with UPEC at the time of inoculation significantly altered acute and chronic UPEC UTI outcomes, despite the fact that GBS was rapidly cleared from the urinary tract within the first 24 hours after infection [3]. This previous study suggests that, in humans, GBS may not be at detectable titers at the time a symptomatic individual visits the clinic despite being present at the time of infection.

Here we extend those studies and show that in virgin nulliparous mice, increased age is associated with *less severe* monomicrobial or polymicrobial acute UTI, in each case characterized by ∼100-fold lower UPEC titers in old vs. young mice. On the other hand, among older mice, multiparity was associated with greater susceptibility to severe high titer acute and chronic UTI. Evidence is presented that this parity-associated augmentation of UTI requires intact inflammatory processes initiated through the microbe-associated pattern receptor TLR4.

## Materials and Methods

### Ethics Statement

All animal experiments were conducted following the National Institutes of Health guidelines for housing and care of laboratory animals and in accordance with institutional regulations and approval by the Committee for Animal Studies at Washington University School of Medicine. During the course of experiments, animal pain and distress was minimized by: performing infections while mice were under isoflurane anesthetic, monitoring them until recovery from isofluorane, and checking the animals again 1–4 hours after infection. Uropathogenic microorganisms do not typically cause severely painful symptoms; nevertheless, mice were closely monitored for signs of pain, distress, or dehydration. While not necessary during our studies, analgesic administration or injectable saline administration via intra peritoneal injection could have been applied for pain or dehydration as needed. At the end of experiments, animals were euthanized by cervical dislocation while under isoflurane anesthetic.

### Bacterial Strains and Growth Conditions

Uropathogenic *E. coli* strain UTI89 [27] or UTI89 *att_HK022_*::*COM-GFP* (kanamycin-resistant, Kan^R^) [28] were inoculated from single colonies grown on LB agar plates into LB containing kanamycin at 25ug/ml where appropriate, and grown statically overnight [18–24 hours (h)] at 37°C as described for infection studies [25], [29], [30], [31] to promote expression of type 1 pili important for bladder infection [32], [33]. *Streptococcus* agalactiae (GBS) wild type strain COH1, a well-characterized strain that expresses low levels of the beta-hemolysin and high levels of the capsular polysaccharide [34], [35], [36], [37] were inoculated from single colonies grown on Todd Hewitt (TH) agar into TH broth (Difco). GBS were grown statically overnight, and then diluted 1∶10 in fresh TH broth for an additional 1 to 2 h at 37°C to an optical density at 600 nm (OD_600_) of approximately 0.4 (logarithmic phase) as previously described for GBS UTI and other *in vivo* virulence studies [3], [4], [5], [38].

### Murine Infections

Bacterial cultures, grown as described above, were collected by centrifugation and resuspended in phosphate-buffered saline (PBS). Female wild-type nulliparous mice aged 7–10 weeks or 8–9 months, or retired breeders (multiparous) ranging from 7–11 months of age were obtained from Harlan (C3H/HeN) or the Jackson Laboratories (C3H/HeJ). Mice were anesthetized by inhalation of 4% isoflurane. Mice were then voided prior to transurethral bacterial inoculation with 1–2×10^7^ CFU in 50 µL [39]. For mixed infections, GBS and UPEC were mixed to obtain a 50 µL bacterial suspension of 1–2×10^7^ CFU of each organism. At indicated time points, mice were euthanized and bladders and kidneys were aseptically removed. The number of bacteria present in the tissues was determined by homogenization of bladders or kidney pairs in PBS and plating of serial dilutions on LB or TH agar supplemented with antibiotics when appropriate. For comparisons of infection in differently aged animals, organ weights of uninfected 7–11 week nulliparous or 8–9 month multiparous animals were measured and used for CFU/g calculations. While most published studies use CFU/organ, we noticed that older mice had significantly larger organs than younger animals. The weight normalization between younger and older mice thus allowed a reduction of the impact of this potential bias on our interpretations. Statistical analyses were performed using the Kruskal-Wallis test with Dunn’s post-test for multiple comparisons where p<0.05 was considered significant with GraphPad Prism software (version 6.00 for Windows, GraphPad Software, www.graphpad.com). Recovered titers of zero were set to the limit of detection of the assay of 40 CFU/organ, and also adjusted for organ weight for comparisons of animals of different ages where appropriate, prior to statistical analyses and graphical representation.

## Results

### Age and Parity are Inversely-related Factors Influencing Acute Cystitis and Ascending Pyelonephritis

**Figure 1:**
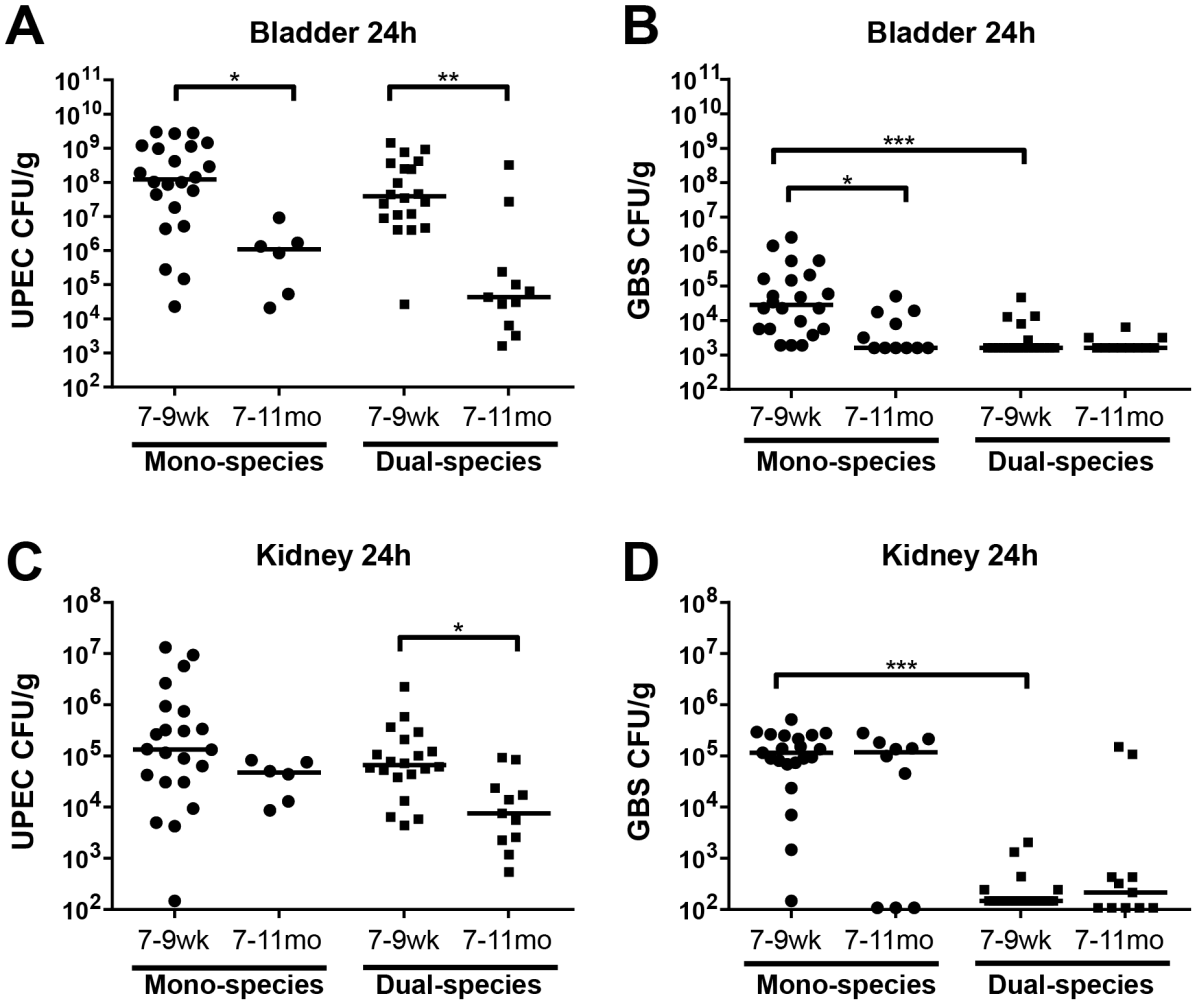
Age-associated risk factors for UTI in nulliparous virgin hosts. Organ weight-adjusted bladder CFU for UPEC (**A**) and GBS (**B**), and kidney CFU for UPEC (**C**) and GBS (**D**) at 24 hours post infection of C3H/HeN female mice with ∼10^7^ UPEC UTI89 alone or with ∼10^7^ GBS COH1 in UPEC+GBS mixed inoculation. N = 1–4, at least 5 mice per experiment, where N indicates the number of biologically distinct experiments performed (N = 4 for 7–9wk groups, N = 1 for UPEC mono-infected 7–11mo group, and N = 2 for GBS mono-infected and all dual-infected 7–11mo groups). Recovered titers of zero were set to the limit of detection of the assay for statistical analyses and graphical representation in all figures. The horizontal bar represents the median value for each group of mice. Statistical significance was determined by the Kruskal-Wallis test with Dunn’s post-test for multiple comparisons. * *P*<0.05, ** *P*<0.005, *** *P*<0.0005.

To evaluate the impact of age, (independent of parity), female mice ranging in age between 7–9 weeks or 7–11 months that had never been bred or given birth (nulliparous virgin) were transurethrally infected with either UPEC or GBS or both. These experiments showed that young nulliparous mice experienced more severe acute cystitis compared to aged nulliparous mice at 24 hours post infection (hpi) (∼100-fold higher bladder titers of UPEC and ∼15-fold higher GBS titers in young vs. aged nulliparous mice) (**Fig. 1A–B**). Younger nulliparous animals also exhibited nearly 1000-fold higher titers of UPEC than aged virgins in the bladder and ∼8-fold higher titers in the kidneys following simultaneous infection with both UPEC and GBS (**Fig. 1A&C**). As described above, bacterial titers in acute GBS cystitis were lower in aged versus young nulliparous virgins (**Fig. 1B**). In contrast, GBS kidney infection was equally robust in older versus younger virgins with approximately 10^5^ GBS recovered from the kidneys in female mice of all ages **(** **Fig. 1D**). UPEC titers were similar in the presence or absence of GBS (**Fig. 1A&C**) whereas, consistent with our previous findings in young mice where we observed that GBS were rapidly cleared after co-infection with UPEC [3], GBS were rapidly cleared when UPEC were also present in the inoculum (**Fig. 1B&D**). CFU/organ data, prior to adjustment for age-associated difference in organ weights, are shown in **Fig. S1**. In summary, these data indicate that, when controlling for parity, increased age was associated with reduced severity of cystitis in general and reduced severity of UPEC pyelonephritis, whereas the severity of GBS pyelonephritis remained constant with increasing age.

**Figure 2:**
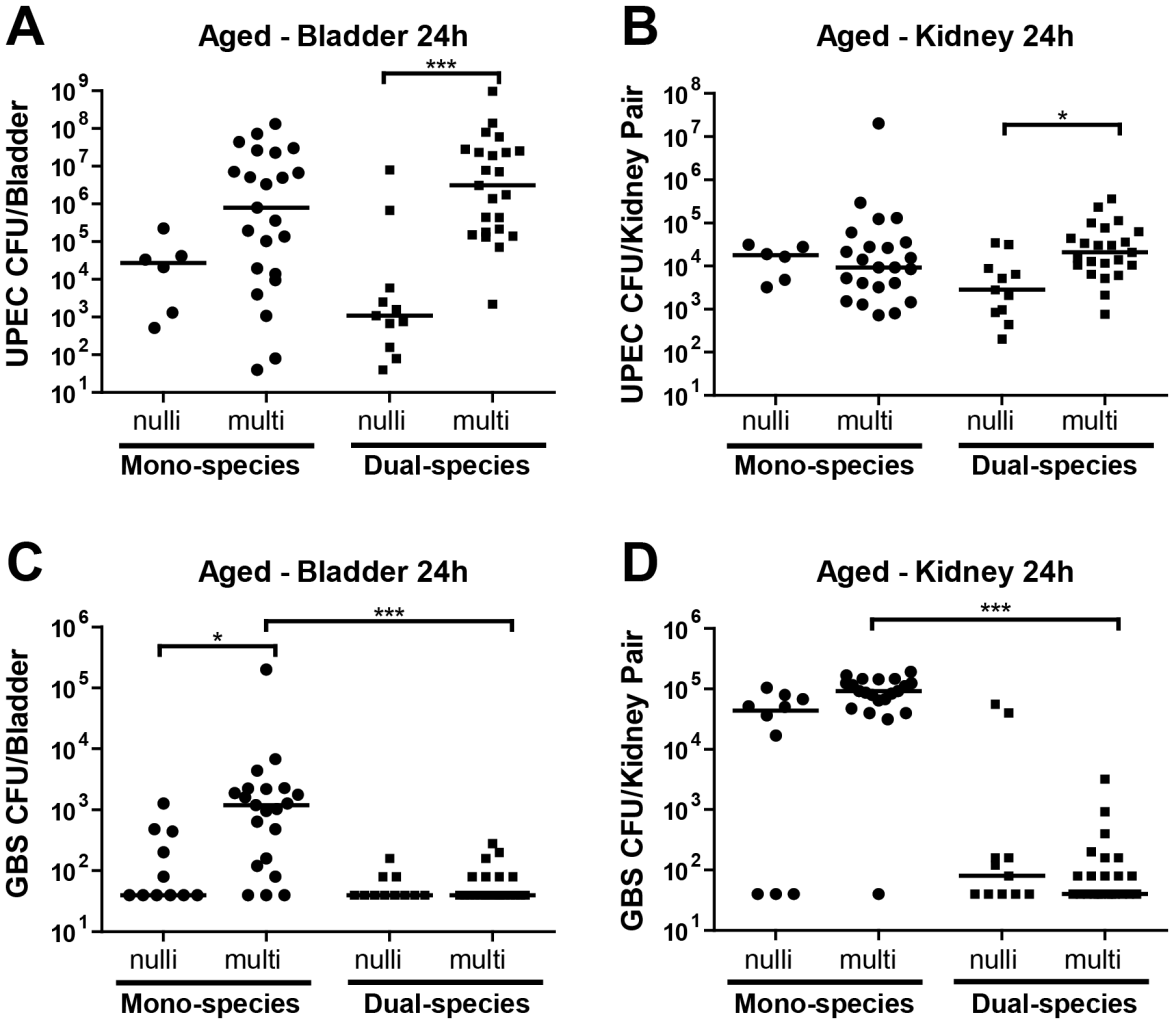
Parity-associated risk factors for UTI in aged hosts. Bladder CFU for UPEC (**A**) and GBS (**C**), and kidney CFU for UPEC (**B**) and GBS (**D**) at 24 hours post infection of C3H/HeN female mice with ∼10^7^ UPEC UTI89 alone or with ∼10^7^ GBS COH1 in UPEC+GBS mixed inoculation. N = 1–4, at least 5 mice per experiment, as described for Fig. 1. Data from 7–11 mo (aged) nulliparous animals are replotted from **Fig. 1** here for ease of comparison with data from aged multiparous hosts. The horizontal bar represents the median value for each group of mice. Statistical significance was determined by the Kruskal-Wallis test with Dunn’s post-test for multiple comparisons. * *P*<0.05, *** *P*<0.0005.

In humans, increasing age is often associated with increasing reproductive experience. Hence, the observations that increased age was associated with decreased UTI severity in **Fig. 1A–D** were unexpected. To further examine the contribution of parity to UTI susceptibility, we compared age-matched nulliparous and multiparous female mice for their susceptibility to UTI. Aged (7–11month old) animals with or without reproductive experience were infected with UPEC, GBS, or co-infected with UPEC+GBS. While there was no significant impact of parity on susceptibility of aged mice to monomicrobial UPEC infection, multiparous mice that were co-infected exhibited nearly 2500-fold higher titers of UPEC in the bladder, and ∼8-fold higher titers of UPEC in the kidney, compared to age-matched nulliparous mice (**Fig. 2A–B** **;** note that the same 7–11 mo nulliparous data are plotted in **Figs. 1** **and** **2** for ease of comparison with data from aged multiparous hosts). Multiparity was also associated with significantly higher GBS CFU in the bladders during GBS mono-species infection (**Fig. 2C–D**). GBS CFU were significantly lower after co-infection with UPEC, compared to GBS mono-infection, albeit only in multiparous mice (**Fig. 2C–D**). Hence, the effects of parity on GBS titers that we observed following mono-species UTI were not detectable in the presence of UPEC.

Together these observations demonstrate that 1) among nulliparous virgin mice, increased age rendered animals less susceptible to acute UTI, and 2) in aged mice, multiparity dramatically increased susceptibility to acute high titer cystitis, particularly in the setting of polymicrobial UTI.

### Multi-parity-augmented UPEC Bladder Infection is TLR4-dependent

**Figure 3:**
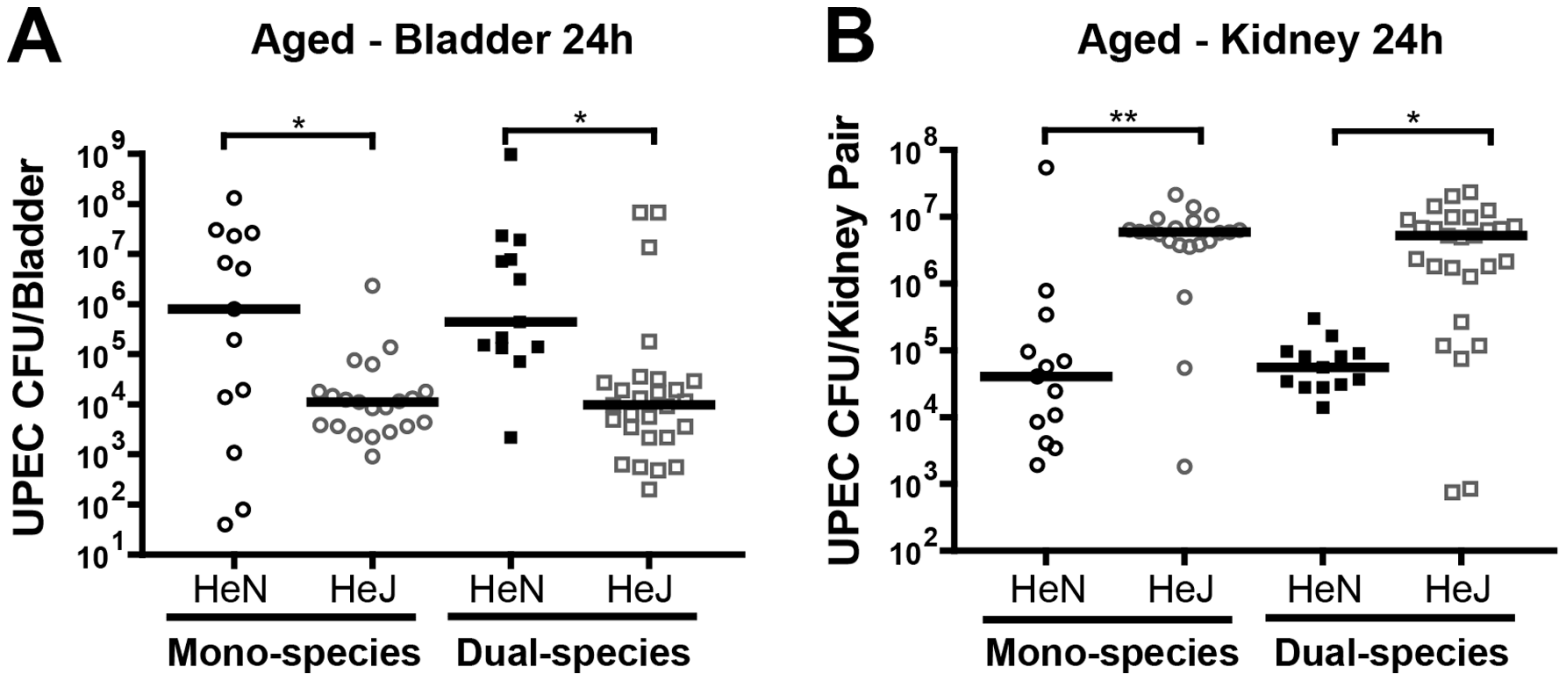
Multiparity-associated augmentation of UPEC cystitis is blunted in C3H/HeJ mice. UPEC CFU in bladders (**A**) and kidneys (**B**) were determined 24 hours after mono- or dual-species infection in 7–11 month old multiparous C3H/HeN and C3H/HeJ mice. N = 2–4, at least 5 mice per experiment. The horizontal bar represents the median value for each group of mice. Statistical significance was determined by the Kruskal-Wallis test with Dunn’s post-test for multiple comparisons. * *P*<0.05, ** *P*<0.005.

Previous studies have shown that TLR4 signaling is required to mount a host response that limits UPEC infection in the bladder [22], [40], [41], [42], [43]. C3H/HeJ mice are unable to mount a TLR4-driven response [44], [45] and exhibit higher titer acute bladder and kidney infections compared to their TLR4 nonresponsive C3H/HeJ counterparts [46]. To examine the contribution of TLR4 signaling in multiparity-augmented UTI, we infected 7–11 month old multiparous C3H/HeJ or C3H/HeN mice with UPEC or with UPEC+GBS and enumerated the CFU in bladders and kidneys at 24 hpi. As described in **Fig. 2A**, multiparous C3H/HeN mice exhibited ∼2500-fold higher titer UPEC cystitis (median 1.25×10^8^ CFU) following dual-species infection compared to their nulliparous counterparts. Surprisingly, after the same dual-species infection in multiparous C3H/HeJ mice, bladder titers were nearly 50-fold lower than in age-matched C3H/HeN mice (**Fig. 3A**). We also observed significantly higher titer cystitis after mono-species UPEC infections in C3H/HeN mice compared to C3H/HeJ mice (**Fig. 3A**). In contrast, aged C3H/HeJ mice had significantly higher titer kidney infections after mono- or dual-species UPEC infections (**Fig. 3B**). These results show that the augmentation of bladder infection in multiparous mice is limited in the absence of TLR4 signaling, strongly suggesting that TLR4-driven inflammation is required for parity-associated susceptibility to severe high titer acute cystitis but not pyelonephritis.

### Multiparity Significantly Increases Susceptibility to Chronic UPEC Infection

**Figure 4:**
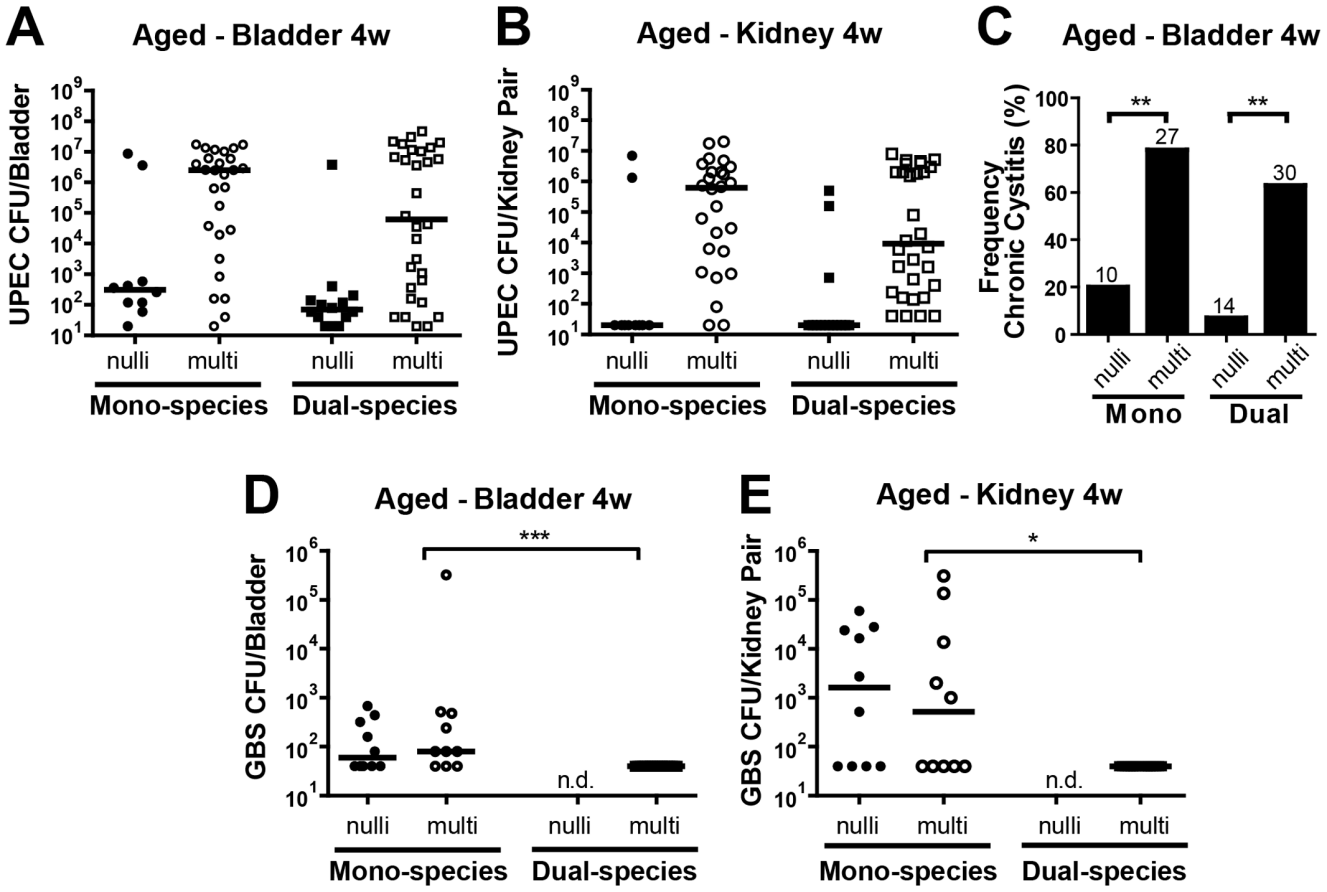
Multiparity is associated with more severe chronic UPEC UTI. Bacterial CFU in the bladder (**A,D**) or kidney (**B,E**) at 4 weeks post infection following infection of C3H/HeN 7–11 month female mice with ∼10^7^ UPEC alone, GBS alone, or ∼10^7^ of each organism simultaneously. For CFU analysis, N = 3–8, at least 5 mice per experiment. The horizontal bar represents the median value for each group of mice. Statistical significance was determined by the Kruskal-Wallis test with Dunn’s post-test for multiple comparisons. * *P*<0.05, ** *P*<0.005, *** *P*<0.0005. N.d. indicates groups in which no data were collected. (**C**) The percentage of mice displaying >10^4^ CFU/bladder at 4 wpi, indicative of chronic cystitis. The number of mice (n) for each infection group is indicated above the bar. Fisher’s Exact test was used for statistical analysis.

We have previously shown that the presence of UPEC and GBS together at the time of urinary tract inoculation rendered young, nulliparous mice significantly more likely to develop chronic bladder infection [3]. Since multiparity was associated with higher UPEC titers during acute UTI, we hypothesized that chronic infection outcomes may be similarly impacted. To test this hypothesis, we infected older (7–11mo) nulli- and multiparous mice with UPEC alone or UPEC+GBS in a dual species inoculation and measured CFUs in bladders and kidneys at 4 weeks post-infection. Similar to the results of *acute* UTI, multiparous animals were dramatically more susceptible to *chronic* cystitis, harboring ∼8000-fold more UPEC CFU in the bladder after single species infection (albeit not statistically significant) and ∼900-fold higher after dual-species infection, compared to nulliparous mice (**Fig. 4A**). In the kidneys, median UPEC CFU after mono- or dual- species infection were at or below the limit of detection in older nulliparous mice at 4 weeks post infection (wpi), but were ∼10^6^ and ∼10^4^ in multiparous mice, respectively (**Fig. 4B**). High UPEC titer (>10^4 ^CFU) in the bladder at 4 wpi is a hallmark of chronic cystitis characterized by high bacterial load in the bladder lumen [19]. The frequency of chronic cystitis (i.e. mice with bladder titers >10^4 ^CFU/mL) was significantly higher in multiparous mice after both mono- and dual-species infection (∼80% vs 20% and 60% vs. 10%, respectively, **Fig. 4C**). We previously showed that GBS is cleared from the bladder of young mice within 2 weeks post infection, but could persist in the kidney in most animals [4]. The current data now demonstrates that most aged mice (irrespective of parity) cannot clear GBS infections from the bladder or kidney (**Fig. 4D–E**).

In summary, we demonstrate that aged multiparous mice exhibit heightened susceptibility to 1) acute high titer GBS cystitis and pyelonephritis, 2) acute high titer UPEC cystitis and pyelonephritis in the context of a polymicrobial exposure containing GBS, and 3) chronic high titer UPEC infection in the presence or absence of GBS.

## Discussion

UTI is a significant public health problem that can affect persons in all age groups; however, host susceptibility to severe, chronic, recurrent, or complicated UTI, as well as the bacterial uropathogen(s) causing UTI, varies over the female lifespan. Our understanding of UTI susceptibility at different life stages is limited, and progress is hampered by a lack of relevant model systems. Here we model UTI susceptibility across a portion of the female lifespan, examining acute and chronic UTI outcomes as a function of bacterial etiology, age, and prior reproductive experience.

Advanced age is associated with an increased risk of a variety of infections in humans, including acute mono-species and polymicrobial UTI [8], [18], [47], [48]. UTI in older adults is attributed to many interrelated factors that include deteriorating immune function, diminished integrity of anatomical barriers, exacerbating underlying medical conditions, altered sex hormone levels, and changes in vaginal microbial ecology [6], [49], [50]. For example, post-menopausal women experience more recurrent chronic UTI than their pre-menopausal counterparts, and lack of estrogen is thought to contribute to this increased susceptibility [51], [52], [53]. However, there are conflicting reports regarding the effect of estrogen therapy on UTI susceptibility in this context [54], [55], [56], [57]. Similar to the human studies, findings in a murine model of surgical menopause are also conflicting. Several studies show that ovariectomized mice exhibited higher bacterial titers during acute cystitis or more severe bladder symptoms [58], [59], and estrogen supplementation of ovariectomized animals limited some UTI-associated symptoms [59]. However another study showed that estrogen treatment resulted in an increased bacterial load in the kidneys [60]. *In vitro* studies showing that estradiol exposure simultaneously improves antimicrobial innate responses and enhances bacterial invasion into urothelial cells may, in part, explain differential effects of estrogen on UTI and discrepancies observed *in vivo* [58].

Among older women, parity has been reported as a risk-factor for UTI in some [7], but not all [61] studies. It is unclear whether many studies with negative results were adequately powered to examine parity-related differences since there are far fewer older nulliparous than multiparous women in most populations. Further, there are some reports of a positive correlation between pregnancy and long-term hormonal effects [62]. For example, one study measuring hormone levels at midcycle and luteal phases of the menstrual cycle in premenopausal women showed increased estradiol levels with increasing age in parous women, while observing reduced estradiol levels with increasing age in nonparous women. [63]. Clearly, the relationships of estradiol levels to age and parity are complex, but this may help to explain the differential host susceptibility to urogenital infection.

Gestation and childbirth are also associated with numerous functional and morphological changes to the urinary tract that may impact UTI susceptibility, including incontinence and genital prolapse [64], [65]. Urine retention is thought to contribute to enhanced UTI susceptibility among elderly and catheterized individuals by limiting bacterial elimination from the bladder and providing a reservoir in which bacteria can proliferate. These physiologic changes have been modeled in rats, where multiparous animals exhibited significantly increased bladder capacity and residual urine volume in the bladder, compared to age-matched nulliparous rats [65], [66].

Surprisingly, and contrary to our initial hypothesis, we found that older nulliparous mice were *less* susceptible to UPEC, GBS, and polymicrobial UTI than were young nulliparous mice. We show that multiparity, rather than increased age rendered C3H/HeN mice more susceptible to acute high titer infections of the bladder (GBS, and UPEC in the presence of GBS), suggesting that the presence of GBS within a polymicrobial inoculum can exacerbate acute UTI outcomes in this context. Based on this finding, future clinical studies should examine GBS as a potential risk factor for acute UPEC UTI in susceptible populations such as pregnant and multiparous elderly women. We also show that parity-associated increased severity of UPEC cystitis was aided by TLR4- signaling in the bladder but not the kidney, since C3H/HeJ mice deficient for TLR4-signaling have less severe cystitis. These data suggest that bladder inflammation specifically enhances UTI in older multiparous mice and we speculate that the UPEC pathogenic cascade within the bladder, and the contribution of inflammation, is significantly different in older multiparous hosts. Further, multiparity significantly enhanced the frequency of UPEC chronic cystitis (in the presence or absence of GBS), which was often accompanied by chronic high titer pyelonephritis. Overall these data firmly establish that multiparity increases susceptibility to UTI in the C3H/HeN model. Further studies are required, including an analysis of sex hormones in the aged nulli- versus multi-parous mice, to clarify if this model can be used to model infection human menopause. These findings also call for further experiments to determine the reason underlying increased susceptibility to UTI in multiparous animals, including parameters of host immune response, and how this may translate into an improved understanding of UTI susceptibility in older at-risk women.

## Supporting Information

Figure S1 **Age-associated risk factors for UTI in nulliparous virgin hosts.** Raw data corresponding to Figure 1, in CFU/organ, are shown.
(TIF)
