# Supplementary figures and images for: Impact of Host Age and Parity on Susceptibility to Severe Urinary Tract Infection in a Murine Model
Source: PLoS One. 2014 May 16;9(5):e97798. doi: 10.1371/journal.pone.0097798 (PMC4024022; doi:10.1371/journal.pone.0097798)

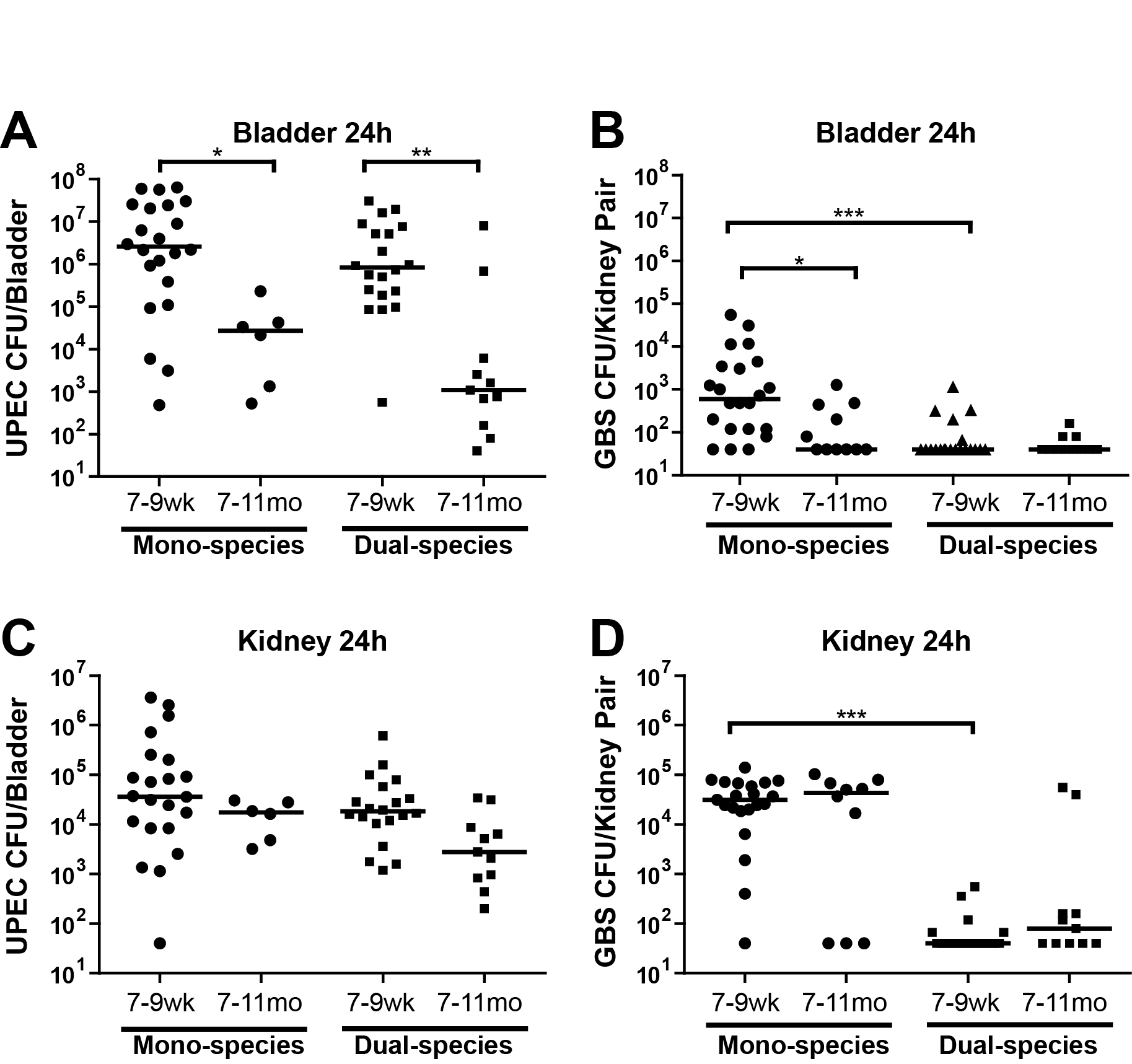

Supplement: Figure S1 — Age-associated risk factors for UTI in nulliparous virgin hosts. Raw data corresponding to Figure 1, in CFU/organ, are shown. (TIF) [file pone.0097798.s001.tif]
